# Blood Biomarkers and the Risk of Coronary Disease in Atrial Fibrillation

**DOI:** 10.1161/JAHA.125.045735

**Published:** 2026-04-20

**Authors:** Andres Cordova Sanchez, Katherine Wilkinson, Samuel A. P. Short, Virginia J. Howard, Suzanne E. Judd, Parag Goyal, Elsayed Z. Soliman, Emily B. Levitan, Monika M. Safford, Mary Cushman

**Affiliations:** ^1^ University of Vermont Larner College of Medicine Burlington VT USA; ^2^ UNC Health Chapel Hill NC USA; ^3^ University of Alabama at Birmingham Birmingham AL USA; ^4^ Weill Cornell Medicine New York NY USA; ^5^ Wake Forest School of Medicine Winston‐Salem NC USA

**Keywords:** atrial fibrillation, biomarkers, coronary heart disease, inflammation, risk factors, Cardiovascular Disease, Atrial Fibrillation, Coronary Artery Disease

## Abstract

**Background:**

Atrial fibrillation (AF) is the most common arrhythmia. Although it was recently identified as a risk factor for coronary heart disease (CHD), the underlying pathophysiology is not well understood. Biomarkers could provide insights on underlying mechanisms and identify potential predictors of CHD among people with AF.

**Methods:**

The REGARDS (Reasons for Geographic and Racial Differences in Stroke) cohort study enrolled 30 239 White and Black adults aged 45 and older in 2003 to 2007. Among those with baseline AF and no history of stroke or CHD, 13 cardiovascular risk biomarkers were measured at baseline. We calculated hazard ratios (HR) for incident CHD by biomarkers using Cox proportional hazards model adjusting for risk factors and use of statins, aspirin, and anticoagulants.

**Results:**

Among 1818 participants with prevalent AF mean age was 69 years (SD 10 years). There were 201 (11%) cases of incident CHD over 9 years. Several biomarkers were associated with incident CHD: NT‐proBNP (N‐terminal pro‐B‐type natriuretic peptide), GDF‐15 (growth differentiation factor 15), C‐reactive protein, interleukin‐6, D‐dimer, cholesterol, lipoprotein(a), triglycerides, low‐density lipoprotein, and gamma‐glutamyl transferase, with HRs 1.17 to 1.69 per SD increment. There were no associations for Factor VIII, galectin 3, and high‐density lipoprotein. The largest associations were for NT‐proBNP and GDF‐15 with respective HRs per SD 1.69 (95% CI, 1.42–2.01) and 1.45 (95% CI, 1.21–1.74). Comparing the top versus bottom tertile, the largest associations were NT‐proBNP (HR 3.14 [95% CI 2.03–4.84]), interleukin‐6 (HR, 2.69 [95% CI, 1.78–4.06]), and GDF‐15 (HR, 2.20 [95% CI, 1.38–3.51]).

**Conclusions:**

Multiple biomarkers were associated with incident CHD in AF with the largest associations being myocardial strain and inflammation.

**Nonstandard Abbreviations and Acronyms**
GDF‐15: growth differentiation factor 15
GGT: gamma‐glutamyl transferase
ln: log‐transformed
Lp(a): lipoprotein (a)
REGARDS: Reasons for Geographic and Racial Differences in Stroke

**Research Perspective**

**What Is New?**

- This study highlights the multifactorial causes of coronary heart disease in atrial fibrillation by providing information on associations of several biomarkers and coronary heart disease risk in this population. The strongest associations were observed for NT‐proBNP (N‐terminal pro‐B‐type natriuretic peptide), GDF‐15 (growth differentiation factor 15), and interleukin‐6.

**What Question Should Be Addressed Next?**

- Can these biomarkers of myocardial strain and inflammation play a role in clinical practice to tailor prevention strategies to reduce coronary heart disease in individuals with atrial fibrillation?

Atrial fibrillation (AF) is the most common arrhythmia worldwide, affecting >10 million people in the United States alone.^1^, ^2^, ^3^ Although AF is a risk factor for coronary heart disease (CHD),^4^ the pathophysiological mechanisms and predictors remain unclear. Weaker associations among those taking anticoagulants suggested a prothrombotic or embolic mechanism.^4^ A proinflammatory state conferred by AF may also be involved.^5^, ^6^ To address these questions, O’Neal et al. studied associations of CRP (C‐reactive protein), D‐dimer, Factor VIII, and fibrinogen with CHD incidence in a small sample of participants with AF, finding a significant association only for D‐dimer.^7^ Several substudies from clinical trial populations also analyzed biomarkers and risk of myocardial infarction in anticoagulated patients with AF,^8^, ^9^, ^10^, ^11^ an important distinction as anticoagulation could potentially affect these associations.^4^

To address the possibility of embolism as cause of increased CHD in AF,^4^, ^7^ Soliman et al. studied whether the association was larger for ST‐segment–elevation myocardial infarction (MI) than non–ST‐segment–elevation MI and found no difference,^12^ challenging this hypothesis. Further study of the associations of different biomarkers and CHD in AF could help identify potential pathophysiologic mechanisms and provide insight on novel prevention strategies. We analyzed the associations of an expanded number of biomarkers with incident CHD in a sample of >1800 people with AF from a national cohort study. We selected biomarkers based on their known associations with risk of cardiovascular disease in general population samples, covering domains of lipid metabolism, liver function, hypercoagulability, myocardial strain, and inflammation.^13^, ^14^, ^15^, ^16^, ^17^, ^18^, ^19^, ^20^, ^21^, ^22^, ^23^, ^24^

## Methods

### Data Sharing Statement

REGARDS (Reasons for Geographic and Racial Differences in Stroke) data are not publicly available due to ethical and legal restrictions. To abide by its obligations with National Institutes of Health/National Institute of Neurological Disorders and Stroke and the Institutional Review Board of the University of Alabama at Birmingham, REGARDS facilitates data sharing through data use agreements. Any investigator is welcome to access the REGARDS data, including statistical code, through this process. Requests for data access may be sent to regardsadmin@uab.edu.

### Study Population

This analysis included participants of the REGARDS study, a prospective cohort of 30 239 White and Black adults aged ≥45 years old. REGARDS was designed to investigate disparities in stroke mortality across the United States and between Black and White individuals. Enrollment occurred between 2003 and 2007 and intentionally oversampled Black individuals and residents of the stroke belt. Participants were approached via mail followed by a telephone interview for medical history and an in‐home visit. The in‐home exam included venipuncture, physical measurements, ECG, and medication inventory. REGARDS had 2 visits approximately 10 years apart and biannual telephone follow‐up to ascertain clinical outcomes. The study design is published elsewhere.^25^ All participants provided verbal and written informed consent. The institutional review boards of the participating institutions approved the study.

We included REGARDS participants with AF and no prior stroke at baseline that was previously described.^26^ AF was defined by self‐report of a physician diagnosis or ECG. These methods were equal predictors of stroke in REGARDS.^27^ We excluded participants with prevalent CHD (defined as self‐reported MI, coronary artery bypass graft, bypass, angioplasty, or stenting or evidence of MI via ECG). The baseline for this analysis was either visit 1 for those with AF detected at visit 1 or visit 2 for those with AF first detected at visit 2. All covariates were taken from the visit at which AF was detected.

### Covariates

Self‐reported age, sex, race, smoking status, and region of residence were used. Race was understood as a social construct representing various social influences that may act as potential confounders, rather than a biological variable. Smoking status was categorized as never, past, or current. Systolic blood pressure was defined as the average of 2 measures in mm Hg. Hypertension status was defined as systolic blood pressure ≥130 mm Hg, diastolic blood pressure ≥80 mm Hg, or self‐reported antihypertensive use. Diabetes was defined as either fasting glucose ≥126 mg/dL, nonfasting glucose ≥200 mg/dL, or self‐reported use of glucose lowering pills or insulin. Use of statins, aspirin, and anticoagulation was assessed by examining pill bottles.

### Laboratory Methods

Details of blood sample handling and assay procedures are published elsewhere^26^, ^28^, ^29^ and provided in Supplemental Methods. Blood samples were retrieved from storage for analysis to correspond with the visit at which AF was first detected.^28^ The 13 biomarkers, selected because they relate to cardiovascular risk and mechanisms^13^, ^14^, ^15^, ^16^, ^17^, ^18^, ^19^, ^20^, ^21^, ^22^, ^23^, ^24^ were total cholesterol, high‐density lipoprotein (HDL), triglycerides, Lp(a) (lipoprotein (a)), calculated low density lipoprotein (LDL), D‐dimer, CRP, Factor VIII antigen, GGT (gamma‐glutamyl transferase), IL‐6 (interleukin‐6), NT‐proBNP (N‐terminal pro‐B type natriuretic peptide), galectin 3, and GDF‐15 (growth differentiation factor 15).

### Outcome Ascertainment

Participants are contacted via telephone every 6 months and medical records were obtained for reported cardiovascular hospitalizations. Two independent adjudicators reviewed all hospitalizations to adjudicate CHD; in case of disagreement the cases were adjudicated by committee review.

CHD ascertainment methods in REGARDS were published elsewhere.^30^ CHD was defined as definite or probable MI or fatal CHD. Definite MI was presence of diagnostic enzymes (troponin or CK‐MB [creatine kinase‐MB] rising or falling over 6 hours or more with a peak 2 times higher the upper limit of normal) or ECG changes consisted with MI based on previously published criteria.^31^ Probable MI was defined as elevated but not diagnostic enzymes with positive but nondiagnostic ECG, or if enzymes were missing with a positive ECG in the presence of ischemic signs or symptoms. Fatal CHD was determined using medical history, hospital records, interviews with next of kin or proxies, and death certificate or National Death Index data. For hospitalized deaths, fatal CHD was defined as death within 28 hours of admission with definitive or probable MI, postmortem findings of MI, or death within 6 hours of admission with cardiac symptoms or signs in the absence of other confirmatory data. For nonhospitalized deaths, fatal CHD was defined as sudden cardiac death, definitive or probable MI within 28 days and no evidence to suggest other cause of death, postmortem evidence of MI <28 days old, history of CHD with cardiac pain up to 72 hours before and no evidence of a different cause of death, or postmortem evidence of chronic CHD.

CHD outcomes in REGARDS are updated annually. The censoring date for this study was December 31, 2020.

### Statistical Analysis

We excluded participants with incomplete covariate data. Nonnormally distributed continuous variables were presented as medians with interquartile ranges, normally distributed continuous variables were presented as mean±SD and categorical variables as counts with percentages. Crude incidence rates of CHD (per 1000 person‐years) were calculated across tertiles of each biomarker.

Biomarkers were modeled as SD increment of the natural log‐transformed (ln) value, tertiles, and restricted cubic splines with 5 knots. For each biomarker considered separately, we calculated hazard ratios (HRs) with 95% CIs of incident CHD using sequential Cox proportional hazards model. Model 1 was unadjusted. Model 2 adjusted for age, sex, race, and region of residence. Model 3 added tobacco use, total cholesterol, HDL, systolic blood pressure, hypertension treatment, diabetes, and the use of statins, aspirin, and anticoagulation. Total cholesterol and HDL were incorporated as they are traditional CHD risk factors. To evaluate their specific associations with CHD in AF, we conducted an additional analysis by excluding both total cholesterol and HDL from Model 3 and adding each one individually, similar to the approach used for other biomarkers. When analyzing triglycerides and LDL, Model 3 was further modified to exclude total cholesterol, as both lipoproteins contribute to total cholesterol levels. The above modeling was repeated for fatal and nonfatal CHD considered separately as well as in subgroups based on anticoagulation status and interaction testing by anticoagulant use. The per‐SD (ln‐transformed), tertile, and spline analyses of biomarkers were prespecified, but the Model 3 per‐SD approach was designated as the primary analysis for interpretation. The proportional hazards assumption was assessed by visual inspection of survival curves and by examining Schoenfeld residuals.

Considering that kidney function may influence biomarker concentrations, and body mass index can affect CHD risk factors, in sensitivity analyses we created new Models 4 and 5 for the per‐SD ln‐transformed biomarker models, we included all the variables from Model 3 and added estimated glomerular filtration rate (Model 4) and body mass index (Model 5).

Statistical significance was defined as 95% CIs that do not cross the null value. Analyses were performed using R (version 4.4.1).

## Results

### Participants

Supplemental Figure 1 shows the cohort selection process. From 30 239 participants enrolled during visit 1, 1,445 had AF without past stroke or CHD. From 16 150 participants at visit 2, 503 had newly detected AF without past stroke or CHD. After excluding participants with missing covariates or follow‐up, the final sample included 1818 participants.

### Baseline Characteristics

Baseline characteristics of the 1818 participants are summarized in Table 1. The mean age was 69, 33% were Black, 57% female, 76% had hypertension, and 21% diabetes; 23% were taking an anticoagulant, with most participants using warfarin. The median follow up was 7.5 years (interquartile range, 5.2–14.1). CHD occurred in 201 participants (80 fatal, 121 nonfatal).

**Table 1:** Baseline Characteristics of 1818 REGARDS Participants With Atrial Fibrillation*

Characteristic	Overall n=1818	No incident CHD n=1617	Incident CHD n=201
Age, y	69 (10)	58 (10)	71 (10)
Race			
White	1227 (67%)	1096 (68%)	131 (65%)
Black	591 (33%)	521 (32%)	70 (35%)
Sex			
Female	1032 (57%)	931 (58%)	101 (50%)
Male	786 (43%)	686 (42%)	100 (50%)
Region			
Nonbelt	807 (44%)	723 (45%)	84 (42%)
Belt	1011 (56%)	894 (55%)	117 (58%)
Smoking			
Past	777 (43%)	684 (42%)	93 (46%)
Never	845 (46%)	758 (47%)	87 (43%)
Current	196 (11%)	175 (11%)	21 (10%)
Systolic blood pressure, mm Hg	125 (117, 136)	124 (116, 136)	129 (119, 141)
Hypertension	1382 (76%)	1205 (75%)	177 (88%)
Hypertension medication use	1087 (62%)	942 (60%)	145 (73%)
Diabetes	387 (21%)	328 (20%)	59 (29%)
Aspirin use	620 (34%)	546 (34%)	74 (37%)
Statin use	597 (33%)	527 (33%)	70 (35%)
Anticoagulation use	417 (23%)	370 (23%)	47 (23%)
Warfarin use	366 (20%)	322 (20%)	44 (22%)

Belt=North Carolina, South Carolina, Georgia, Alabama, Tennessee, Arkansas, and Louisiana. CHD indicates coronary heart disease.

* Results are presented as mean±SD, median (interquartile range) or frequency (%).

### Biomarkers and CHD

**Figure 1:**
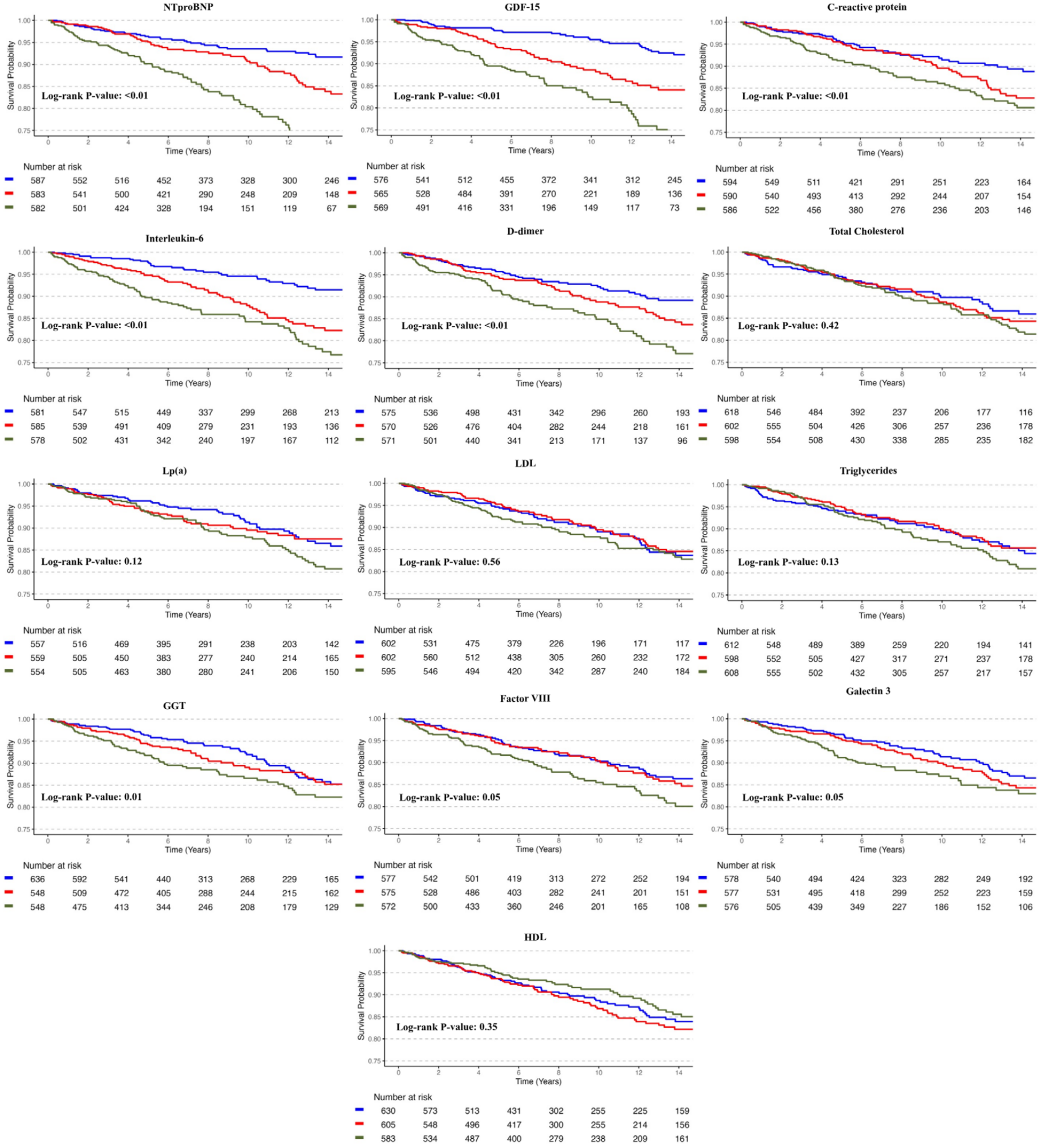
Kaplan–Meier analysis of incident CHD by tertiles of each biomarker in REGARDS participants with baseline AF. Blue=Tertile 1. Red=Tertile2. Green=Tertile 3. *Tertile cut‐ points per biomarker*: NT‐proBNP (74.37, 269.4 pg/mL); GDF‐15 (934.6, 1416.1 pg/mL); C‐reactive protein (1.4, 4.21 mg/L); interleukin‐6 (0.84, 1.57 pg/mL); D‐dimer (0.36, 0.65 μg/mL FEU); total cholesterol (167, 201 mg/dL); Lp(a) (9, 33 mg/dL); triglycerides (89, 137 mg/dL); LDL (91, 119 mg/dL); GGT (17, 27 IU/L); Factor VIII (112.25, 141.87%); galectin‐3 (10.29, 13.77 ng/mL); and HDL (44, 58 mg/dL). CHD indicates coronary heart disease; GDF‐15, growth differentiation factor 15; GGT, gamma‐glutamyl transferase; HDL, high‐density lipoprotein; LDL, low density lipoprotein; Lp(a), lipoprotein(a); NT‐proBNP, N‐terminal pro–B‐type natriuretic peptide; and REGARDS, Reasons for Geographic and Racial Differences in Stroke.

Median and interquartile range concentrations of each biomarker are displayed in Supplemental Table 1. Kaplan–Meier curves for time to CHD are shown in Figure 1. Incidence rates per 1000 person‐years and incidence rate ratios by tertile of each biomarker are shown in Supplemental Table 2. The highest incidence rates of CHD in the top tertile of biomarkers were for NT‐proBNP and GDF‐15, with incidence rates of 21.7 and 20.1 per 1000 person‐years for the highest tertiles, respectively, and incidence rate ratios >3 compared with the bottom tertile.

**Figure 2:**
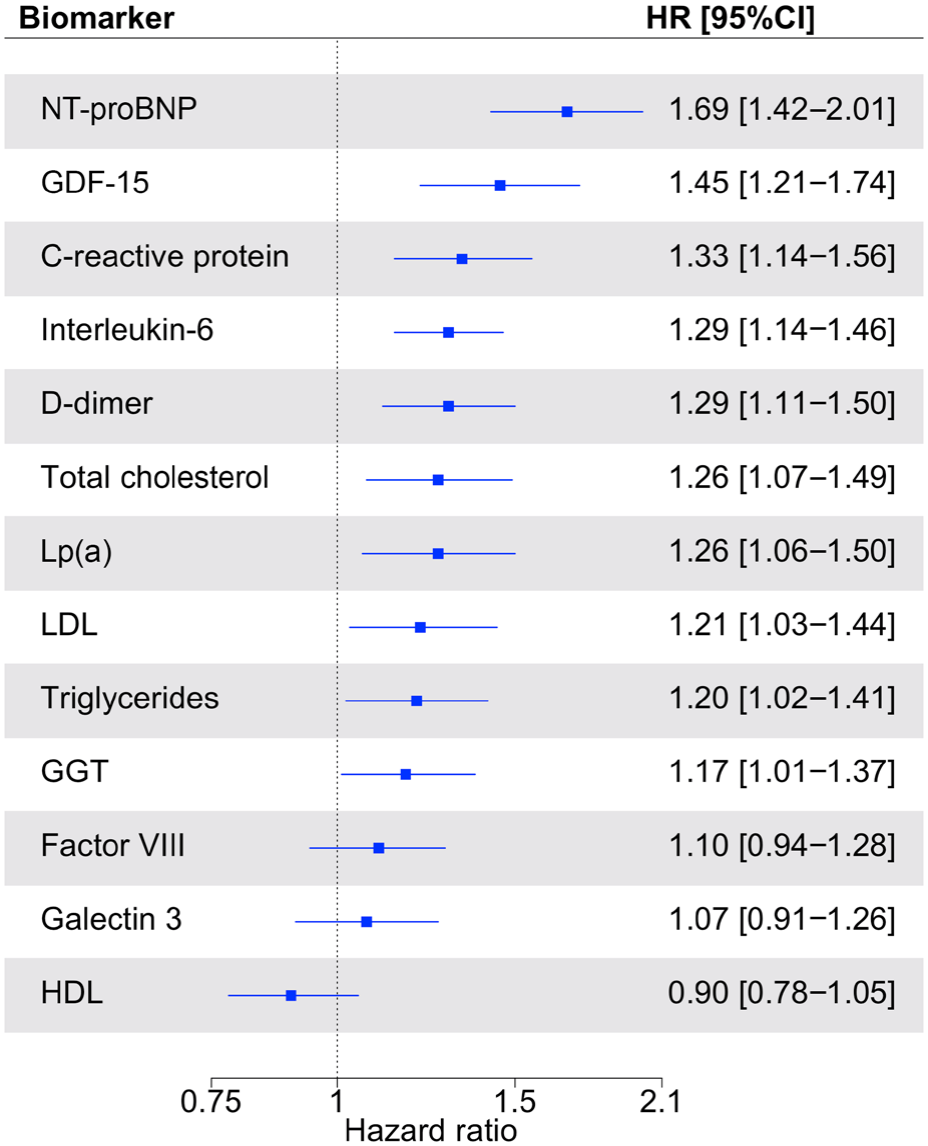
Adjusted hazard ratios of CHD per SD increment of ln‐transformed biomarkers in REGARDS participants with baseline atrial fibrillation. Each biomarker was modeled separately. HRs are adjusted for age, sex, race, region of residence, tobacco use, total cholesterol, HDL, systolic blood pressure, hypertension treatment, diabetes, and use of statins, aspirin, and warfarin. To analyze total cholesterol and HDL, each was eliminated from the model during its respective analysis. When analyzing triglycerides and LDL, total cholesterol was excluded, as both lipoproteins contribute to total cholesterol levels. CHD indicates coronary heart disease; GDF‐15, growth differentiation factor 15; GGT, gamma‐glutamyl transferase; HLD, high‐density lipoprotein; HR, hazard ratio; LDL, low density lipoprotein; LP(a), lipoprotein(a); and NTproBNP, N‐terminal pro–B‐type natriuretic peptide; and REGARDS, Reasons for Geographic and Racial Differences in Stroke.

Associations of each biomarker with incident CHD per ln‐transformed SD increment are shown in Figure 2 for Model 3 and Supplemental Table 3 for Models 1 and 2. In the unadjusted model (Model 1), significant associations were observed for NT‐proBNP, GDF‐15, CRP, IL‐6, D‐dimer, Lp(a), LDL, triglycerides, GGT, Factor VIII, and galectin 3. In Models 2 and 3, the associations of Factor VIII and galectin 3 became nonsignificant, whereas total cholesterol became significantly associated with CHD. For all other biomarkers across the 3 model adjustments, associations were modestly attenuated except CRP, Lp(a), triglycerides, LDL, and GGT, where no attenuation was observed. In Model 3, considered as the HR per SD increment of the ln of each biomarker, the highest risk of CHD was seen with NT‐proBNP (HR, 1.69 [95% CI, 1.42–2.01]) and GDF‐15 (HR, 1.45 [95% CI, 1.21–1.74]). Significant associations were also found for CRP, IL‐6, D‐dimer, total cholesterol, Lp(a), LDL, triglycerides, and GGT With HRs ranging from 1.17 to 1.33. There were no major differences in HR after adjusting for kidney function and body mass index (Supplemental Table 3).

Considering biomarkers as tertiles, Model 3 results are shown in Table 2 and results of other models can be seen in Supplemental Table 4. Comparing the second and third tertiles with the first tertile, risk of CHD increased for higher NT‐proBNP, GDF‐15, and IL‐6. For CRP, D‐dimer, total cholesterol, LDL, and GGT, the association was present only for the third versus the first tertile. The largest associations for the top versus bottom tertile of biomarkers were for NT‐proBNP (HR, 3.14 [95% CI, 2.03–4.84]), IL‐6 (HR, 2.69 95% CI, 1.78–4.06]) and GDF‐15 (HR, 2.20 [95% CI, 1.38–3.55]). Associations for most biomarkers appeared linear in spline models (Supplemental Figure 2).

**Table 2:** Adjusted Hazard Ratios of Incident CHD by Tertiles of Biomarkers in REGARDS Participants With Baseline Atrial Fibrillation*

	HR CHD (95% CI)	
Biomarker	Tertile 2 vs 1†	Tertile 3 vs 1
N‐terminal pro–B‐type natriuretic peptide	1.61 (1.07–2.43)	3.14 (2.03–4.84)
Growth differentiation factor 15	1.63 (1.05–2.54)	2.20 (1.38–3.51)
C‐reactive protein	1.25 (0.86–1.83)	1.94 (1.32–2.84)
Interleukin‐6	1.80 (1.19–2.73)	2.69 (1.78–4.06)
D‐dimer	1.14 (0.78–1.67)	1.50 (1.01–2.23)
Total cholesterol	1.39 (0.96–2.02)	1.94 (1.30–2.88)
Lipoprotein(a)	1.09 (0.74–1.62)	1.44 (0.96–2.17)
LDL	1.20 (0.83–1.74)	1.60 (1.09–2.35)
Triglycerides	0.92 (0.64–1.34)	1.23 (0.83–1.81)
Gamma‐glutamyl transferase	1.10 (0.76–1.60)	1.63 (1.14–2.35)
Factor VIII	0.88 (0.61–1.26)	1.11 (0.78–1.60)
Galectin 3	1.11 (0.76–1.61)	1.23 (0.84–1.81)
HDL	1.15 (0.82–1.74)	0.84 (0.58–1.23)

When analyzing triglycerides and LDL, total cholesterol was excluded, as both lipoproteins contribute to total cholesterol levels.

* Adjusted for age, sex, race, region of residence, included tobacco use, total cholesterol, HDL, systolic blood pressure, hypertension treatment, diabetes, and use of statins, aspirin, and anticoagulation. To analyze total cholesterol and HDL, each was eliminated from model 3 during its respective analysis.

^†^ Tertile cut‐points are shown in Figure 1.

CHD indicates coronary heart disease; HDL, high‐density lipoprotein; LDL, low density lipoprotein; and REGARDS, Reasons for Geographic and Racial Differences in Stroke.

No significant violations of the proportional hazard assumptions were observed for the global model 3 or for individual biomarkers except for triglycerides, which showed a marginal deviation with a slight increase in association late in follow up (*P* for Schoenfeld residual=0.03). Visual inspection of the Schoenfeld residual plots supported overall proportionality.

Results of analysis for fatal and nonfatal CHD are shown in Supplemental Table 5. Each SD increment of NT‐proBNP, GDF‐15, IL‐6, and D‐dimer was associated with both fatal and nonfatal CHD. Total cholesterol, Lp(a), LDL, and GGT were associated with only nonfatal CHD. NT‐proBNP had a substantial association with fatal CHD (HR, 2.36 [95% CI, 1.80–3.09]).

Biomarker associations stratified by anticoagulation status are shown in Supplemental Table 6. Interaction testing for anticoagulation was significant for NT‐proBNP and CRP. For NT‐proBNP the HR per SD increment was 1.67 (95% CI, 1.39–2.00) for those not on anticoagulation and 1.23 (95% CI, 0.87–1.73) for individuals who were on anticoagulants; *P* interaction=0.05. For CRP these HRs were 1.26 (95% CI, 1.05–1.51) and 1.82 (95% CI, 1.32–2.52) respectively; *P* interaction=0.05.

## Discussion

In this study of ~2000 participants with AF from a general population sample, multiple biomarkers were associated with incident CHD. The strongest associations were for higher NT‐proBNP, GDF‐15, CRP, and IL‐6. There were no associations for Factor VIII, galectin 3, triglycerides, and HDL. Associations of higher NT‐proBNP were weaker in those on versus not on anticoagulation, whereas this pattern was opposite for CRP and not different for other biomarkers.

Other studies assessed the associations between biomarkers and CHD in AF.^10^, ^11^, ^32^, ^33^ O’Neal et al. analyzed a convenience sample of ∼800 REGARDS participants with AF and examined 4 biomarkers related to inflammation and coagulation: CRP, D‐dimer, Factor VIII, and fibrinogen. In 4.4 years of follow‐up, only D‐dimer was associated with incident CHD.^7^ Compared with the O’Neal study the current analysis was more robust by including more than twice as many participants, assessing a broader panel of 13 biomarkers across multiple physiological domains, and having median follow‐up of 7.5 years. Several clinical trial substudies biomarkers such as NT‐proBNP, GDF‐15, IL‐6, CRP, and troponins with CHD in anticoagulated patients with AF. These found associations between inflammatory biomarkers and CHD death but not myocardial infarction, and no association of NT‐proBNP and CHD.^8^, ^9^, ^10^, ^11^, ^32^, ^33^ Recently, Meyre et al. published a study of 12 biomarkers (including D‐dimer, GDF‐15, IL‐6, and NT‐proBNP) and myocardial infarction among Swiss patients with AF seen in clinics. Higher IL‐6, GDF‐15, NT‐proBNP, and high‐sensitivity troponin T were associated with myocardial infarction (HR for all, ~1.2 per SD increment). In this study, patients were enrolled in clinics, 84% were receiving anticoagulation and patients with unrecognized AF were not included.^34^ In contrast to the study by Meyre et al. and the subanalyses of clinical trials, we included a general population sample of people in the United States irrespective of anticoagulation status. Both clinical trial participants and patients seen in clinics can differ from the general population. Similarly, anticoagulation could affect biomarker associations.^4^

To provide context to the current findings, we compared our results with studies in general populations; associations with CHD among people with AF generally appeared stronger (Table 3). REGARDS lacks comparable biomarker data in participants without AF, so we compared findings to the literature. Based on the larger associations among those with AF, it seems possible that these findings could inform future research on CHD risk prediction and prevention in AF.

**Table 3:** Comparison of CHD Risk by Biomarker Levels in Atrial Fibrillation Participants in REGARDS Versus Studies of General Population Samples

Biomarker	AF participants in REGARDS	General population studies	References
N‐terminal pro–B‐type natriuretic peptide	1.69 (1.42–2.01)	1.22 (1.09–1.38) 1.30 (0.90–1.80)* 1.77 (1.30–2.41)*	Pareek 2017^60^ Bibbins‐Domingo 2007^15^ Mishra 2014^36^
Growth differentiation factor 15	1.45 (1.21–1.74)	1.24 (1.19–1.30)*	Kato 2023^14^
C‐reactive protein	1.33 (1.14–1.56)	1.10 (0.99–1.23) 1.16 (1.11–1.22)*	Pareek 2017^60^ Mani 2019^19^
Interleukin‐6	1.29 (1.14–1.46)	1.13 (1.02–1.25)	Pareek 2017^60^
D‐dimer	1.29 (1.11–1.50)	1.05 (1.00–1.11) 1.27 (1.11–1.45)^†^	Folsom 2016^61^ Zakai^‡^ 2017^16^
Total cholesterol	1.26 (1.07–1.49)	1.22 (1.19–1.25)	Peters 2016^13^
Lipoprotein(a)	1.26 (1.06–1.50)	1.26 (1.02–1.56)*	Colantonio‡ 2022^21^
Low‐density lipoprotein	1.21 (1.03–1.44)	1.10 (1.05–1.17)	Zakai‡ 2022^24^
Triglycerides	1.20 (1.02–1.41)	1.05 (1.01–1.1)	Zakai‡ 2022^24^
Gamma‐glutamyl transferase	1.17 (1.01–1.37)	1.13 (1.03–1.24)	Lee 2007^20^
Factor VIII	1.10 (0.94–1.28)	1.52 (1.29–1.79)	Zakai‡ 2018^17^
Galectin 3	1.07 (0.91–1.26)	1.30 (1.06–1.60)	Aguilar 2020^22^
High‐density lipoprotein	0.90 (0.78–1.05)	0.95 (0.89–1.02)	Zakai‡ 2022^24^

Hazard ratios per SD increment are shown unless indicated. Bolded characters=Results from the overall REGARDS population.

AF indicates atrial fibrillation; CHD, coronary heart disease; HR, hazard ratio; and REGARDS, Reasons for Geographic and Racial Differences in Stroke.

* Risk of CHD per SD in patients with underlying coronary artery disease or atherosclerotic cardiovascular disease, no studies of the general population that could be reliably compared with the current study were found.

^†^ HR represents risk per doubling of biomarker.

^‡^ If available, REGARDS data were presented to allow comparison from the same study.

NT‐proBNP had the largest association with CHD in AF; comparing the top versus bottom tertile the HR was 3.14 (95% CI, 2.03–4.84) and the HR per SD increment was 1.69 (95% CI, 1.42–2.01). NT‐proBNP is associated with recurrent CHD regardless of heart failure diagnosis in those with stable coronary disease.^15^, ^35^, ^36^, ^37^ In AF, others have reported higher NT‐proBNP was associated with higher risk of CHF, cardiovascular death, and stroke.^9^, ^26^, ^38^, ^39^, ^40^ Here, CHD risk in AF was similar to that of patients with underlying stable coronary disease irrespective of AF diagnosis.^15^ The association of NT‐proBNP was attenuated by anticoagulation use, consistent with clinical trial subanalyses and the study by Meyre et al., which did not show an association between NTproBNP and CHD risk in predominantly anticoagulated individuals with AF.^9^, ^10^, ^34^ In our study, the risk was higher for fatal CHD at 236% per SD increment. Our findings suggest that NT‐proBNP not only predicts CHD risk but also identifies individuals at high risk of fatal events.

Given the proinflammatory state conferred by AF^5^, ^41^, ^42^, ^43^, ^44^ and the long‐known link between inflammation and CHD,^45^ we expected inflammatory biomarkers might play a greater role in CHD risk among individuals with AF than in the general population. GDF‐15, an emerging biomarker associated with incident AF, stroke, diabetes, metabolic syndrome, and CHD,^11^, ^14^, ^32^, ^46^, ^47^, ^48^ had the strongest association with CHD in AF among the studied inflammatory markers. The HR of CHD per SD increment in GDF‐15 (HR, 1.45 [95% CI, 1.21–1.74]) was numerically higher than past reports in the general population (HR, 1.24 [95% CI, 1.19–1.3).^14^ Though CIs, overlap and no direct comparison between participants with and without AF was made. In the current study, CRP and IL‐6 were associated with 33% and 29% per SD increment risk of CHD respectively; these findings for GDF‐15, CRP, and IL‐6 were consistent with those presented by Meyre et al.^34^ Unlike our study, the O’Neal study did not find an association between CRP and CHD,^7^ likely explained by our larger sample and longer follow‐up. Compared with people with established heart disease, the associations of CRP and IL‐6 with CHD were higher in our study (33% and 29% increase per SD versus 16% and 23% increase per SD respectively).^18^, ^19^ Clinical trial subanalyses in anticoagulated individuals with AF found associations between elevated GDF‐15, IL‐6, and CRP with cardiac death but not with myocardial infarction,^11^, ^32^ possibly due to shorter follow‐up, lower power, or participant differences between clinical trial and general populations. Further studies are needed to determine whether inflammatory biomarkers can improve CHD risk prediction in AF and whether anti‐inflammatory treatments could help reduce this risk.

Embolic MI from left atrial thrombus may contribute to CHD in AF.^4^, ^7^ AF also confers a prothrombotic state via endothelial dysfunction and increased coagulation activity, which may also promote CHD.^6^, ^49^ A previous REGARDS study found that individuals with AF on anticoagulation had a similar CHD risk as the general population (HR, 0.76 [95% CI, 0.29–1.94]) whereas those not on anticoagulation had an increased risk (HR, 1.92; 95% CI 1.42–2.60).^4^ Our findings for D‐dimer and CHD risk in AF align with the O’Neal study from REGARDS (HR, 1.29 [95% CI, 1.11–1.5]). Similarly, Factor VIII showed no association with CHD in AF.^7^ To compare with prior studies reporting a 1.27‐fold CHD risk increase per D‐dimer doubling in the general population,^16^ we performed the same calculation and found a similar CHD risk in AF at 1.25 (95% CI, 1.1–1.4). Meyre et al. did not find an association with D‐dimer and CHD in their cohort of predominantly anticoagulated patients with AF.^34^ These findings suggest that the role of D‐dimer in CHD risk is not AF specific. Future research should examine whether other markers of coagulation and thrombosis contribute to CHD in AF and whether antiplatelet or anticoagulation medications influence CHD risk based on biomarker levels.

The association between dyslipidemia and CHD was established in the early 1900s.^50^ We analyzed total cholesterol, calculated LDL, Lp(a), and triglycerides, with the largest associations with CHD in AF for total cholesterol and Lp(a), each with a 26% increased risk of CHD per SD increase. The association of total cholesterol with CHD here in participants with AF was similar to those in the general population.^13^ By contrast, the associations of LDL and triglycerides with CHD in AF appeared larger than in the overall REGARDS population; each SD increment of LDL and triglycerides corresponded to a ~20% increase in CHD in participants with AF versus 10% and 5% respectively in the overall REGARDS cohort.^24^ A similar association was seen in fully anticoagulated patients with elevated apolipoprotein B from the ARISTOTLE (Apixaban for the Prevention of Stroke in Subjects With Atrial Fibrillation) trial.^8^ Lp(a) is of particular interest in AF given its prothrombotic and proinflammatory properties^6^, ^51^, ^52^ and potential as a novel therapeutic target.^53^ In our analysis, Lp(a) was associated with a 26% increased risk of CHD per SD increment, similar to past reports in participants with baseline cardiovascular disease.^21^

The associations of total cholesterol, LDL, and triglycerides with CHD in AF, along with the low percentage of REGARDS participants with AF taking statins at baseline (33%), raises questions regarding the potential for improved CHD prevention through lipid‐lowering therapies in this population. The use of statins decreases CHD incidence in the general population by approximately 30%.^54^, ^55^ In AF, statin use is associated with lower cardiovascular mortality and stroke.^56^ However, its effects on CHD in this population are unknown. Considering our findings, this is a high priority area for further research.

There is conflicting evidence regarding the association between GGT and CHD in the general population,^57^, ^58^, ^59^ possibly because GGT could be a marker of metabolic syndrome more so than of CHD. However, some evidence suggests that GGT has a direct effect in CHD.^58^ Here, GGT showed 17% increased risk of CHD per SD increment among participants with AF. Although we did not adjust for all components of metabolic syndrome, our findings highlight the need for further studies to elucidate GGT’s role in CHD among those with AF.

We explored whether anticoagulation modified the associations between these biomarkers and CHD risk. CRP had a stronger association in participants on anticoagulants compared with those not on anticoagulation, suggesting inflammation may play a role in CHD development and risk prediction among individuals with AF on anticoagulants. Although NT‐proBNP demonstrated the strongest association with CHD, this relationship was attenuated by anticoagulation use, suggesting that higher NT‐proBNP might predict a benefit of anticoagulation to prevent CHD in AF.

### Limitations and Strengths

This study has limitations. AF diagnosis was either self‐reported or identified by ECG at baseline. Recall bias may have caused misclassification, especially for nonpermanent AF. However, we can be reasonably confident that REGARDS participants with AF actually had AF.^27^ Despite adjusting for several covariates, unmeasured confounding is possible. As with most epidemiology studies we were limited to a single measurement of biomarkers, so misclassification is possible. Due to the observational study design, we are unable to establish causal relationships. REGARDS included only Black and White individuals within the United States, limiting generalizability. These findings have not been replicated in an external cohort. Unfortunately, we did not have comparable biomarker data in REGARDS participants without AF, so we were unable to make direct comparisons. Future research should formally assess whether the associations of biomarkers with CHD differ by AF status. This is a hypothesis‐generating study focused on biological insights into CHD risk in AF; therefore, we did not measure the clinical utility of biomarkers for CHD risk prediction. Future research is needed to understand roles for risk prediction tools for CHD specifically in AF and to address whether these biomarkers might have a role in guiding clinical management. We did not have information on type of AF or MI (type 1 versus type 2) and could not assess the potential variability in biomarker relevance across AF or MI subtypes.

Several strengths of this study should be pointed out. REGARDS is a large, high‐quality, longitudinal study that includes Black and White individuals across the United States. Blood samples and biomarker measurements were handled with standardized methods, and we used commercially available assay kits. There was a high retention rate (97% annually) and expert adjudication of medical records was used to classify CHD incidence.

## Conclusions

We provide new insight into the mechanisms contributing to the elevated risk of CHD in AF. The associations of various biomarkers with CHD highlight a multifactorial nature of this risk, involving myocardial strain, inflammation, lipid metabolism, and hemostasis. Translational research should address the causative nature of these findings and interventions that might be effective in risk reduction. Future research is needed to assess these biomarkers in risk prediction of CHD in AF and to explore their potential for guiding clinical management and therapeutic strategies.

## Sources of Funding

This research project is supported by RF1 NS041588 co‐funded by the National Institute of Neurological Disorders and Stroke (and the National Institute on Aging, National Institutes of Health, Department of Health and Human Service. Additional support was from the National Institute of General Medical Sciences, P20 GM135007 (Mary Cushman), the American Heart Association Student Research Award in Cerebrovascular Disease and Stroke (Samuel A. P. Short), and the National Heart Lung and Blood Institute R01 HL80477 and R01 HL165452. This content is the sole responsibility of the authors and does not necessarily represent the official views of the National Institute of Neurological Disorders and Stroke or the National Institutes of Health.

## Disclosures

Emily Levitan receives research funding from American Heart Association, and Amgen Inc and personal fees for serving on a data and safety monitoring boardfrom the University of Pittsburgh. All other authors do not have conflicts of interest related to this article.

## Supporting information

Data S1
Tables S1–S6
Figures S1–S2

## References

[1] Noubiap JJ , Tang JJ , Teraoka JT , Dewland TA , Marcus GM . Minimum national prevalence of diagnosed atrial fibrillation inferred from California acute care facilities. J Am Coll Cardiol. 2024;84:1501–1508. doi: 10.1016/j.jacc.2024.07.014 39269390

[2] Colilla S , Crow A , Petkun W , Singer DE , Simon T , Liu X . Estimates of current and future incidence and prevalence of atrial fibrillation in the U.S. adult population. Am J Cardiol. 2013;112:1142–1147. doi: 10.1016/j.amjcard.2013.05.063 23831166

[3] Martin SS , Aday AW , Allen NB , Almarzooq ZI , Anderson CAM , Arora P , Avery CL , Baker‐Smith CM , Bansal N , Beaton AZ , et al. 2025 heart disease and stroke statistics: a report of US and global data from the American Heart Association. Circulation. 2025;151:e41–e660. doi: 10.1161/CIR.0000000000001303 39866113 PMC12256702

[4] Soliman EZ , Safford MM , Muntner P , Khodneva Y , Dawood FZ , Zakai NA , Thacker EL , Judd S , Howard VJ , Howard G , et al. Atrial fibrillation and the risk of myocardial infarction. JAMA Intern Med. 2014;174:107–114. doi: 10.1001/jamainternmed.2013.11912 24190540 PMC4115282

[5] Guo Y , Lip GY , Apostolakis S . Inflammation in atrial fibrillation. J Am Coll Cardiol. 2012;60:2263–2270. doi: 10.1016/j.jacc.2012.04.063 23194937

[6] Khan AA , Lip GYH . The prothrombotic state in atrial fibrillation: pathophysiological and management implications. Cardiovasc Res. 2019;115:31–45. doi: 10.1093/cvr/cvy272 30388199

[7] O’Neal WT , Soliman EZ , Howard G , Howard VJ , Safford MM , Cushman M , Zakai NA . Inflammation and hemostasis in atrial fibrillation and coronary heart disease: the REasons for geographic and racial differences in stroke study. Atherosclerosis. 2015;243:192–197. doi: 10.1016/j.atherosclerosis.2015.09.009 26398291 PMC4634936

[8] Pol T , Held C , Westerbergh J , Lindback J , Alexander JH , Alings M , Erol C , Goto S , Halvorsen S , Huber K , et al. Dyslipidemia and risk of cardiovascular events in patients with atrial fibrillation treated with Oral anticoagulation therapy: insights from the ARISTOTLE (Apixaban for Reduction in Stroke and Other Thromboembolic Events in Atrial Fibrillation) trial. J Am Heart Assoc. 2018;7:e007444. doi: 10.1161/JAHA.117.007444 29419390 PMC5850246

[9] Hijazi Z , Wallentin L , Siegbahn A , Andersson U , Christersson C , Ezekowitz J , Gersh BJ , Hanna M , Hohnloser S , Horowitz J , et al. N‐terminal pro‐B‐type natriuretic peptide for risk assessment in patients with atrial fibrillation: insights from the ARISTOTLE trial (Apixaban for the Prevention of Stroke in Subjects With Atrial Fibrillation). J Am Coll Cardiol. 2013;61:2274–2284. doi: 10.1016/j.jacc.2012.11.082 23563134

[10] Hijazi Z , Oldgren J , Andersson U , Connolly SJ , Ezekowitz MD , Hohnloser SH , Reilly PA , Vinereanu D , Siegbahn A , Yusuf S , et al. Cardiac biomarkers are associated with an increased risk of stroke and death in patients with atrial fibrillation: a randomized evaluation of long‐term anticoagulation therapy (RE‐LY) substudy. Circulation. 2012;125:1605–1616. doi: 10.1161/CIRCULATIONAHA.111.038729 22374183

[11] Hijazi Z , Aulin J , Andersson U , Alexander JH , Gersh B , Granger CB , Hanna M , Horowitz J , Hylek EM , Lopes RD , et al. Biomarkers of inflammation and risk of cardiovascular events in anticoagulated patients with atrial fibrillation. Heart. 2016;102:508–517. doi: 10.1136/heartjnl-2015-308887 26839066

[12] Soliman EZ , Lopez F , O’Neal WT , Chen LY , Bengtson L , Zhang ZM , Loehr L , Cushman M , Alonso A . Atrial fibrillation and risk of ST‐segment‐elevation versus non‐ST‐segment‐elevation myocardial infarction: the atherosclerosis risk in communities (ARIC) Study. Circulation. 2015;131:1843–1850. doi: 10.1161/CIRCULATIONAHA.114.014145 25918127 PMC4447576

[13] Peters SA , Singhateh Y , Mackay D , Huxley RR , Woodward M . Total cholesterol as a risk factor for coronary heart disease and stroke in women compared with men: a systematic review and meta‐analysis. Atherosclerosis. 2016;248:123–131. doi: 10.1016/j.atherosclerosis.2016.03.016 27016614

[14] Kato ET , Morrow DA , Guo J , Berg DD , Blazing MA , Bohula EA , Bonaca MP , Cannon CP , de Lemos JA , Giugliano RP , et al. Growth differentiation factor 15 and cardiovascular risk: individual patient meta‐analysis. Eur Heart J. 2023;44:293–300. doi: 10.1093/eurheartj/ehac577 36303404 PMC10066747

[15] Bibbins‐Domingo K , Gupta R , Na B , Wu AH , Schiller NB , Whooley MA . N‐terminal fragment of the prohormone brain‐type natriuretic peptide (NT‐proBNP), cardiovascular events, and mortality in patients with stable coronary heart disease. JAMA. 2007;297:169–176. doi: 10.1001/jama.297.2.169 17213400 PMC2848442

[16] Zakai NA , McClure LA , Judd SE , Kissela B , Howard G , Safford M , Cushman M . D‐dimer and the risk of stroke and coronary heart disease. The REasons for Geographic and Racial Differences in Stroke (REGARDS) Study. Thromb Haemost. 2017;117:618–624. doi: 10.1160/TH16-07-0519 28004063 PMC5824689

[17] Zakai NA , Judd SE , Kissela B , Howard G , Safford MM , Cushman M . Factor VIII, protein C and cardiovascular disease risk: the REasons for Geographic and Racial Differences in Stroke Study (REGARDS). Thromb Haemost. 2018;118:1305–1315. doi: 10.1055/s-0038-1655766 29890521 PMC6028294

[18] Ridker PM , MacFadyen JG , Glynn RJ , Bradwin G , Hasan AA , Rifai N . Comparison of interleukin‐6, C‐reactive protein, and low‐density lipoprotein cholesterol as biomarkers of residual risk in contemporary practice: secondary analyses from the Cardiovascular Inflammation Reduction Trial. Eur Heart J. 2020;41:2952–2961. doi: 10.1093/eurheartj/ehaa160 32221587 PMC7453833

[19] Mani P , Puri R , Schwartz GG , Nissen SE , Shao M , Kastelein JJP , Menon V , Lincoff AM , Nicholls SJ . Association of Initial and Serial C‐reactive protein levels with adverse cardiovascular events and death after acute coronary syndrome: a secondary analysis of the VISTA‐16 trial. JAMA Cardiol. 2019;4:314–320. doi: 10.1001/jamacardio.2019.0179 30840024 PMC6484785

[20] Lee DS , Evans JC , Robins SJ , Wilson PW , Albano I , Fox CS , Wang TJ , Benjamin EJ , D’Agostino RB , Vasan RS . Gamma glutamyl transferase and metabolic syndrome, cardiovascular disease, and mortality risk: the Framingham Heart Study. Arterioscler Thromb Vasc Biol. 2007;27:127–133. doi: 10.1161/01.ATV.0000251993.20372.40 17095717

[21] Colantonio LD , Bittner V , Safford MM , Marcovina S , Brown TM , Jackson EA , Li M , Lopez JAG , Monda KL , Plante TB , et al. Lipoprotein(a) and the risk for coronary heart disease and ischemic stroke events among black and white adults with cardiovascular disease. J Am Heart Assoc. 2022;11:e025397. doi: 10.1161/JAHA.121.025397 35621195 PMC9238745

[22] Aguilar D , Sun C , Hoogeveen RC , Nambi V , Selvin E , Matsushita K , Saeed A , McEvoy JW , Shah AM , Solomon SD , et al. Levels and change in galectin‐3 and association with cardiovascular events: the ARIC Study. 2020;9:e015405. doi: 10.1161/JAHA.119.015405 PMC7670497 32573308

[23] Yang G , Mason AM , Wood AM , Schooling CM , Burgess S . Dose‐response associations of lipid traits with coronary artery disease and mortality. JAMA Netw Open. 2024;7:e2352572. doi: 10.1001/jamanetworkopen.2023.52572 38241044 PMC10799266

[24] Zakai NA , Minnier J , Safford MM , Koh I , Irvin MR , Fazio S , Cushman M , Howard VJ , Pamir N . Race‐dependent association of high‐density lipoprotein cholesterol levels with incident coronary artery disease. J Am Coll Cardiol. 2022;80:2104–2115. doi: 10.1016/j.jacc.2022.09.027 36423994

[25] Howard VJ , Cushman M , Pulley L , Gomez CR , Go RC , Prineas RJ , Graham A , Moy CS , Howard G . The reasons for geographic and racial differences in stroke study: objectives and design. Neuroepidemiology. 2005;25:135–143. doi: 10.1159/000086678 15990444

[26] Short, S , Hald, E , Wilkinson, K , Howard, G , Howard, VJ , Judd, SE , Soliman, EZ , Kissela, BM , Robinson, D , Stanton, RJ et al Circulating biomarkers and ischemic stroke risk in adults with atrial fibrillation taking anticoagulation: the REGARDS Study. Journal of Thrombosis and Haemostasis 2025;10:3148–3159. doi: 10.1186/s12885-025-15027-6 PMC12931963 40774831

[27] Soliman EZ , Howard G , Meschia JF , Cushman M , Muntner P , Pullicino PM , McClure LA , Judd S , Howard VJ . Self‐reported atrial fibrillation and risk of stroke in the reasons for geographic and racial differences in stroke (REGARDS) study. Stroke. 2011;42:2950–2953. doi: 10.1161/STROKEAHA.111.621367 21817138 PMC3185239

[28] Gillett SR , Boyle RH , Zakai NA , McClure LA , Jenny NS , Cushman M . Validating laboratory results in a national observational cohort study without field centers: the Reasons for Geographic and Racial Differences in Stroke cohort. Clin Biochem. 2014;47:243–246. doi: 10.1016/j.clinbiochem.2014.08.003 25130959 PMC5038129

[29] Cushman M , McClure LA , Howard VJ , Jenny NS , Lakoski SG , Howard G . Implications of increased C‐reactive protein for cardiovascular risk stratification in black and white men and women in the US. Clin Chem. 2009;55:1627–1636. doi: 10.1373/clinchem.2008.122093 19643839 PMC2810186

[30] Safford MM , Brown TM , Muntner PM , Durant RW , Glasser S , Halanych JH , Shikany JM , Prineas RJ , Samdarshi T , Bittner VA , et al. Association of race and sex with risk of incident acute coronary heart disease events. JAMA. 2012;308:1768–1774. doi: 10.1001/jama.2012.14306 23117777 PMC3772637

[31] Prineas RJ , Crow RS , Zhang ZM . The Minnesota Code Manual of Electrocardiographic Findings. Springer‐Verlag; 2010.

[32] Wallentin L , Hijazi Z , Andersson U , Alexander JH , De Caterina R , Hanna M , Horowitz JD , Hylek EM , Lopes RD , Asberg S , et al. Growth differentiation factor 15, a marker of oxidative stress and inflammation, for risk assessment in patients with atrial fibrillation: insights from the Apixaban for Reduction in Stroke and Other Thromboembolic Events in Atrial Fibrillation (ARISTOTLE) trial. Circulation. 2014;130:1847–1858. doi: 10.1161/CIRCULATIONAHA.114.011204 25294786

[33] Hijazi Z , Siegbahn A , Andersson U , Granger CB , Alexander JH , Atar D , Gersh BJ , Mohan P , Harjola VP , Horowitz J , et al. High‐sensitivity troponin I for risk assessment in patients with atrial fibrillation: insights from the Apixaban for Reduction in Stroke and other Thromboembolic Events in Atrial Fibrillation (ARISTOTLE) trial. Circulation. 2014;129:625–634. doi: 10.1161/CIRCULATIONAHA.113.006286 24226808

[34] Meyre PB , Aeschbacher S , Blum S , Reichlin T , Haller M , Rodondi N , Muller AS , Bernheim A , Beer JH , Moschovitis G , et al. Biomarker panels for improved risk prediction and enhanced biological insights in patients with atrial fibrillation. Nat Commun. 2025;16:7042. doi: 10.1038/s41467-025-62218-7 40744929 PMC12313968

[35] Bibbins‐Domingo K , Ansari M , Schiller NB , Massie B , Whooley MA . B‐type natriuretic peptide and ischemia in patients with stable coronary disease: data from the Heart and Soul study. Circulation. 2003;108:2987–2992. doi: 10.1161/01.CIR.0000103681.04726.9C 14662720 PMC2771188

[36] Mishra RK , Beatty AL , Jaganath R , Regan M , Wu AH , Whooley MA . B‐type natriuretic peptides for the prediction of cardiovascular events in patients with stable coronary heart disease: the Heart and Soul Study. J Am Heart Assoc. 2014;3:e000907. doi: 10.1161/JAHA.114.000907 25053234 PMC4310375

[37] Hussain A , Sun W , Deswal A , de Lemos JA , McEvoy JW , Hoogeveen RC , Matsushita K , Aguilar D , Bozkurt B , Virani SS , et al. Association of NT‐ProBNP, blood pressure, and cardiovascular events: the ARIC Study. J Am Coll Cardiol. 2021;77:559–571. doi: 10.1016/j.jacc.2020.11.063 33538254 PMC7945981

[38] Singleton MJ , Yuan Y , Dawood FZ , Howard G , Judd SE , Zakai NA , Howard VJ , Herrington DM , Soliman EZ , Cushman M . Multiple blood biomarkers and stroke risk in atrial fibrillation: the REGARDS Study. J Am Heart Assoc. 2021;10:e020157. doi: 10.1161/JAHA.120.020157 34325516 PMC8475705

[39] Brady PF , Chua W , Nehaj F , Connolly DL , Khashaba A , Purmah YJV , Ul‐Qamar MJ , Thomas MR , Varma C , Schnabel RB , et al. Interactions between atrial fibrillation and natriuretic peptide in predicting heart failure hospitalization or cardiovascular death. J Am Heart Assoc. 2022;11:e022833. doi: 10.1161/JAHA.121.022833 35112889 PMC9245805

[40] Cushman M , Callas PW , McClure LA , Unverzagt FW , Howard VJ , Gillett SR , Thacker EL , Wadley VG . N‐terminal pro‐B‐type natriuretic peptide and risk of future cognitive impairment in the REGARDS cohort. J Alzheimer’s Dis. 2016;54:497–503. doi: 10.3233/JAD-160328 27567834

[41] Rizos I , Rigopoulos AG , Kalogeropoulos AS , Tsiodras S , Dragomanovits S , Sakadakis EA , Faviou E , Kremastinos DT . Hypertension and paroxysmal atrial fibrillation: a novel predictive role of high sensitivity C‐reactive protein in cardioversion and long‐term recurrence. J Hum Hypertens. 2010;24:447–457. doi: 10.1038/jhh.2009.89 20072146

[42] Aviles RJ , Martin DO , Apperson‐Hansen C , Houghtaling PL , Rautaharju P , Kronmal RA , Tracy RP , Van Wagoner DR , Psaty BM , Lauer MS , et al. Inflammation as a risk factor for atrial fibrillation. Circulation. 2003;108:3006–3010. doi: 10.1161/01.CIR.0000103131.70301.4F 14623805

[43] Ederhy S , Di Angelantonio E , Dufaitre G , Meuleman C , Masliah J , Boyer‐Chatenet L , Boccara F , Cohen A . C‐reactive protein and transesophageal echocardiographic markers of thromboembolism in patients with atrial fibrillation. Int J Cardiol. 2012;159:40–46. doi: 10.1016/j.ijcard.2011.02.020 21402418

[44] Marcus GM , Whooley MA , Glidden DV , Pawlikowska L , Zaroff JG , Olgin JE . Interleukin‐6 and atrial fibrillation in patients with coronary artery disease: data from the heart and soul Study. Am Heart J. 2008;155:303–309. doi: 10.1016/j.ahj.2007.09.006 18215601 PMC2247366

[45] Ridker PM , Cushman M , Stampfer MJ , Tracy RP , Hennekens CH . Inflammation, aspirin, and the risk of cardiovascular disease in apparently healthy men. N Engl J Med. 1997;336:973–979. doi: 10.1056/NEJM199704033361401 9077376

[46] Chen M , Ding N , Mok Y , Mathews L , Hoogeveen RC , Ballantyne CM , Chen LY , Coresh J , Matsushita K . Growth differentiation factor 15 and the subsequent risk of atrial fibrillation: the Atherosclerosis Risk in Communities Study. Clin Chem. 2022;68:1084–1093. doi: 10.1093/clinchem/hvac096 35762561

[47] Eddy AC , Trask AJ . Growth differentiation factor‐15 and its role in diabetes and cardiovascular disease. Cytokine Growth Factor Rev. 2021;57:11–18. doi: 10.1016/j.cytogfr.2020.11.002 33317942 PMC7897243

[48] Asrih M , Wei S , Nguyen TT , Yi HS , Ryu D , Gariani K . Overview of growth differentiation factor 15 in metabolic syndrome. J Cell Mol Med. 2023;27:1157–1167. doi: 10.1111/jcmm.17725 36992609 PMC10148061

[49] Ding WY , Gupta D , Lip GYH . Atrial fibrillation and the prothrombotic state: revisiting Virchow’s triad in 2020. Heart. 2020;106:1463–1468. doi: 10.1136/heartjnl-2020-316977 32675218

[50] Kannel WB , Dawber TR , Friedman GD , Glennon WE , McNamara PM . Risk factors in coronary heart disease. An evaluation of several serum lipids as predictors of coronary heart disease; the Framingham Study. Ann Intern Med. 1964;61:888–899. doi: 10.7326/0003-4819-61-5-888 14233810

[51] Boffa MB . Beyond fibrinolysis: the confounding role of Lp(a) in thrombosis. Atherosclerosis. 2022;349:72–81. doi: 10.1016/j.atherosclerosis.2022.04.009 35606079

[52] van der Valk FM , Bekkering S , Kroon J , Yeang C , den Van Bossche J , van Buul JD , Ravandi A , Nederveen AJ , Verberne HJ , Scipione C , et al. Oxidized phospholipids on lipoprotein(a) elicit Arterial Wall inflammation and an inflammatory monocyte response in humans. Circulation. 2016;134:611–624. doi: 10.1161/CIRCULATIONAHA.116.020838 27496857 PMC4995139

[53] Thau H , Neuber S , Emmert MY , Nazari‐Shafti TZ . Targeting lipoprotein(a): can RNA therapeutics provide the next step in the prevention of cardiovascular disease? Cardiol Ther. 2024;13:39–67. doi: 10.1007/s40119-024-00353-w 38381282 PMC10899152

[54] Cholesterol Treatment Trialists C , Mihaylova B , Emberson J , Blackwell L , Keech A , Simes J , Barnes EH , Voysey M , Gray A , Collins R , et al. The effects of lowering LDL cholesterol with statin therapy in people at low risk of vascular disease: meta‐analysis of individual data from 27 randomised trials. Lancet. 2012;380:581–590. doi: 10.1016/S0140-6736(12)60367-5 22607822 PMC3437972

[55] Force USPST , Mangione CM , Barry MJ , Nicholson WK , Cabana M , Chelmow D , Coker TR , Davis EM , Donahue KE , Jaen CR , et al. Statin use for the primary prevention of cardiovascular disease in adults: US preventive services task Force recommendation statement. JAMA. 2022;328:746–753. doi: 10.1001/jama.2022.13044 35997723

[56] Pastori D , Baratta F , Di Rocco A , Farcomeni A , Del Ben M , Angelico F , Violi F , Pignatelli P , Lip GYH . Statin use and mortality in atrial fibrillation: a systematic review and meta‐analysis of 100,287 patients. Pharmacol Res. 2021;165:105418. doi: 10.1016/j.phrs.2021.105418 33450384

[57] Stranges S , Dorn JM , Muti P , Freudenheim JL , Farinaro E , Russell M , Nochajski TH , Trevisan M . Body fat distribution, relative weight, and liver enzyme levels: a population‐based study. Hepatology. 2004;39:754–763. doi: 10.1002/hep.20149 14999694

[58] Giral P , Ratziu V , Chapman JC . Letter regarding article by Ruttmann et al, “gamma‐Glutamyltransferase as a risk factor for cardiovascular disease mortality: an epidemiological investigation in a cohort of 163,944 Austrian adults”. Circulation. 2006;113:e299; author reply e299‐300. doi: 10.1161/CIRCULATIONAHA.105.594176 16505183

[59] Ruttmann E , Brant LJ , Concin H , Diem G , Rapp K , Ulmer H ; Vorarlberg Health Monitoring and Promotion Program Study Group . Gamma‐glutamyltransferase as a risk factor for cardiovascular disease mortality: an epidemiological investigation in a cohort of 163,944 Austrian adults. Circulation. 2005;112:2130–2137. doi: 10.1161/CIRCULATIONAHA.105.552547 16186419

[60] Pareek M , Bhatt DL , Vaduganathan M , Biering‐Sorensen T , Qamar A , Diederichsen AC , Moller JE , Hindersson P , Leosdottir M , Magnusson M , et al. Single and multiple cardiovascular biomarkers in subjects without a previous cardiovascular event. Eur J Prev Cardiol. 2017;24:1648–1659. doi: 10.1177/2047487317717065 28644092

[61] Folsom AR , Gottesman RF , Appiah D , Shahar E , Mosley TH . Plasma d‐dimer and incident ischemic stroke and coronary heart disease: the atherosclerosis risk in communities Study. Stroke. 2016;47:18–23. doi: 10.1161/STROKEAHA.115.011035 26556822 PMC4696899

