# Supplementary material for: Blood Biomarkers and the Risk of Coronary Disease in Atrial Fibrillation
Source: J Am Heart Assoc. 2026 Apr 20;15(9):e45735. doi: 10.1161/JAHA.125.045735 (PMC13279119; doi:10.1161/JAHA.125.045735)
Supplement: Supplementary file 1 — Data S1 Tables S1–S6 Figures S1–S2 [file JAH3-15-e45735-s001.pdf]

Supplemental Material

Methods
Laboratory Procedures:

Blood samples were obtained using standardized methods, centrifuged for 10 minutes within 120 minutes of phlebotomy, and then shipped on ice, recentrifuged at high speed, and stored at -80 °C at the University of Vermont Laboratory for Clinical Biochemistry Research (LCBR).[28] The examined biomarkers were total cholesterol, high-density lipoprotein (HDL), triglycerides, lipoprotein (a) [Lp(a)], calculated low density lipoprotein (LDL), D-dimer, CRP, factor VIII antigen, gamma-glutamyl transferase (GGT), interleukin-6 (IL-6), N-terminal pro-B type natriuretic peptide (NTproBNP), galectin 3, and growth differentiation factor 15 (GDF-15). They were assayed from blood samples obtained at each participant's baseline (visit 1 or visit 2) as previously reported.[26]

Assays were performed at the LCBR using commercially available kits. Lp(a) and CRP were measured using particle-enhanced immunonephelometry on a BNII nephelometer (formerly Dade Behring now Siemens AG, Munich, Germany)[29]. Lipid profile was quantified with colorimetric reflectance spectrophotometry, utilizing the Ortho Vitros Clinical Chemistry System 950IRC (Johnson & Johnson Clinical Diagnostics, Rochester, NY)[29]. D-dimer was measured by the Evolution automated coagulation analyzer, using an immuno-turbidometric assay (Liatest D-DI; Diagnostica Stago, Parsippany, NJ). Factor VIII antigen was measured using enzyme immunoassay kits from Enzyme Research Laboratories (South Bend, IN). Galectin 3, GDF-15, and IL-6 were measured by enzyme immunoassay kits from R&D Systems (Minnesota, USA). GGT was measured using an enzymatic colorimetric assay from Roche (Basel, Switzerland), Finally, NTproBNP was measured by electrochemiluminescence immunoassay on the Roche Elecsys 2010 analyzer (Roche Diagnostics Indianapolis, IN).

Table S1. Blood Biomarker Concentration Overall and by CHD Incidence.

	All<br>N = 1,818<br>Median (25th-75th percentile)	No CHD<br>N = 1,617<br>Median (25th-75th percentile)	CHD<br>N = 201<br>Median (25th-75th percentile)
NTproBNP (pg/mL)	138 (56, 421)	131 (54, 388)	228 (95, 726)
GDF-15 (pg/mL)	1,147 (836, 1,634)	1,121 (824, 1,581)	1,380 (1,012, 1,961)
C-reactive protein (mg/L)	2.44 (1.04, 5.76)	2.36 (1.04, 5.55)	3.33 (1.46, 7.16)
Interleukin-6 (pg/mL)	1.14 (0.72, 1.90)	1.08 (0.71, 1.84)	1.48 (0.91, 2.58)
D-dimer (µg/ml fibrinogen equivalent units)	0.48 (0.32, 0.80)	0.47 (0.31, 0.79)	0.58 (0.36, 1.04)
Factor VIII antigen (%)	126 (105, 153)	126 (105, 152)	127 (106, 156)
Total Cholesterol (mg/dL)	184 (158, 211)	184 (157, 210)	188 (164, 219)
Lp(a) (mg/dL)	18 (6, 47)	17 (6, 46)	24 (8, 59)
LDL (mg/dL)	105 (83, 129)	105 (83, 128)	109 (85, 133)
Triglycerides (mg/dL)	109 (80, 155)	108 (80, 153)	119 (87, 183)
HDL (mg/dL)	50 (41, 63)	50 (41, 63)	49 (40, 60)
GGT (IU/L)	21 (14, 33)	21 (14, 33)	24 (16, 34)
Galectin 3 (ng/mL)	12.0 (9.5, 15.0)	11.9 (9.4, 14.9)	12.7 (9.8, 15.5)

IQR= interquartile range. CHD= coronary heart disease. NTproBNP= N-terminal pro–B-type natriuretic peptide. GDF-15 = growth differentiation factor 15. GGT= Gamma-glutamyl transferase. Lp(a)= lipoprotein (a). HDL= High-density lipoprotein, LDL= low density lipoprotein.

Table S2. CHD Incidence Rates (per 1,000 Person-Years) and Incidence Rate Ratios by Tertile of Biomarker in REGARDS Participants with Baseline Atrial Fibrillation

	**Tertile 1 (reference)**	**Tertile 2**		**Tertile 3**	
	**IR/1,000 PY**	**IR/1,000 PY**	**IRR**	**IR/1,000 PY**	**IRR**
**NTproBNP**	6.6	11.5	1.73	21.7	3.26
**GDF-15**	5.4	12.4	2.30	20.1	3.72
**C-reactive protein**	8.5	11.9	1.39	15.7	1.84
**Interleukin-6**	6.0	12.8	2.12	18.7	3.11
**D-dimer**	8.8	11.6	1.32	17.7	2.02
**TOTAL Cholesterol**	11.2	11.7	1.04	13.7	1.22
**LP(a)**	9.9	10.5	1.06	14.0	1.41
**LDL**	11.7	11.4	0.97	13.5	1.15
**Triglycerides**	11.6	10.6	0.91	14.6	1.26
**GGT**	9.4	11.0	1.17	15.6	1.64
**Factor VIII**	10.5	11.2	1.06	15.6	1.48
**Galectin 3**	9.4	11.3	1.2	14.5	1.54
**HDL**	12.9	13.3	1.03	10.4	0.81

IR= incidence rate. PY= person-years. IRR= incidence rate ratio.
NTproBNP= N-terminal pro–B-type natriuretic peptide. GDF-15 = growth differentiation factor 15. GGT= Gamma-glutamyl transferase. LP(a)= lipoprotein (a). HDL= High-density lipoprotein, LDL= low density lipoprotein.

Table S3. Associations of per SD Increment of ln-transformed Biomarkers with CHD Incidence in REGARDS Participants with Baseline Atrial Fibrillation

**Biomarker**	**HR Incident CHD per SD Increment (95% CI)** *				
	Model 1	Model 2	Model 3	Model 4	Model 5
**NTproBNP**	1.90 (1.62- 2.23)	1.64 (1.37- 1.96)	1.67 (1.39- 2.00)	1.67 (1.39- 2.00)	1.64 (1.36 - 1.96)
**GDF15**	1.82 (1.55- 2.13)	1.54 (1.28- 1.85)	1.46 (1.18- 1.80)	1.45 (1.19- 1.77)	1.43 (1.17 - 1.75)
**C-reactive protein**	1.20 (1.02- 1.41)	1.32 (1.11- 1.58)	1.26 (1.05- 1.51)	1.33 (1.14- 1.56)	1.29 (1.10 - 1.52)
**Interleukin-6**	1.34 (1.18- 1.52)	1.29 (1.13- 1.48)	1.28 (1.11- 1.47)	1.29 (1.14- 1.46)	1.26 (1.11 - 1.43)
**D-dimer**	1.53 (1.33- 1.76)	1.34 (1.14- 1.57)	1.35 (1.14- 1.60)	1.27 (1.09- 1.48)	1.22 (1.04 - 1.43)
**Cholesterol**	1.08 (0.91- 1.28)	1.21 (1.02- 1.43)	1.28 (1.07- 1.55)	1.25 (1.06- 1.48)	1.26 (1.06 - 1.49)
**LP(a)**	1.23 (1.04- 1.46)	1.30 (1.07- 1.59)	1.27 (1.04- 1.55)	1.26 (1.06- 1.50)	1.27 (1.06 - 1.51)
**Triglycerides**	1.16 (1.00- 1.36)	1.27 (1.08- 1.49)	1.23 (1.03- 1.48)	1.21 (1.03- 1.43)	1.20 (1.02 - 1.41)
**LDL**	1.07 (0.90- 1.26)	1.18 (0.99- 1.40)	1.24 (1.02- 1.50)	1.18 (1.01- 1.39)	1.21 (1.02 - 1.43)
**GGT**	1.16 (0.98- 1.37)	1.24 (1.05- 1.47)	1.16 (0.98- 1.38)	1.18 (1.01- 1.37)	1.18 (1.01 - 1.38)
**Factor VIII**	1.16 (1.00- 1.35)	1.10 (0.93- 1.29)	1.08 (0.91- 1.28)	1.07 (0.92- 1.25)	1.05 (0.90 - 1.23)
**Galectin 3**	1.21 (1.01- 1.45)	1.13 (0.95- 1.36)	1.06 (0.88- 1.27)	1.05 (0.88- 1.24)	1.03 (0.87 - 1.22)
**HDL**	0.94 (0.81- 1.11)	0.95 (0.80- 1.11)	0.94 (0.80- 1.12)	0.91 (0.79- 1.06)	0.93 (0.80 - 1.09)

* Model 1 was unadjusted.
Model 2 adjusted for age, sex, race, and region of residence.
Model 3 included tobacco use, total cholesterol, HDL, systolic blood pressure, hypertension treatment, diabetes, and use of statins, aspirin, and anticoagulation.
Model 4 included variables from model 2, model 3, plus estimated glomerular filtration rate.
Model 5 included variables from model 4, plus BMI.
To analyze total cholesterol and HDL, each was eliminated from model 3 during its respective analysis. When analyzing triglycerides and LDL, total cholesterol was excluded, as both lipoproteins contribute to total cholesterol levels.

NTproBNP= N-terminal pro–B-type natriuretic peptide. GDF-15 = growth differentiation factor 15. GGT= Gamma-glutamyl transferase. LP(a)= lipoprotein (a). HDL= High-density lipoprotein, LDL= low density lipoprotein. BMI= Body Mass Index.

Table S4. Associations of Tertiles of Biomarkers with Incident CHD in REGARDS Participants with Baseline Atrial Fibrillation.

		HR CHD (95% CI)*	
**Biomarker**	**Model**	**Tertile 2 vs 1**	**Tertile 3 vs 1**
**NTproBNP**	1	1.77 (1.2-2.63)	3.40 (2.34-4.92)
	2	1.63 (1.09-2.46)	2.65 (1.75-4.01)
	3	1.61 (1.07-2.43)	3.14 (2.03-4.84)
**GDF-15**	1	2.36 (1.55-3.59)	3.89 (2.60-5.82)
	2	1.84 (1.19-2.84)	2.69 (1.73-4.19)
	3	1.63 (1.05-2.54)	2.20 (1.38-3.51)
**C-Reactive Protein**	1	1.40 (0.96-2.03)	1.85 (1.29-2.66)
	2	1.36 (0.93-1.98)	2.23 (1.55-3.23)
	3	1.25 (0.86-1.83)	1.94 (1.32-2.84)
**Interleukin-6**	1	2.14 (1.43-3.21)	3.15 (2.13-4.65)
	2	1.93 (1.28-2.90)	2.88 (1.94-4.29)
	3	1.80 (1.19-2.73)	2.69 (1.78-4.06)
**D-Dimer**	1	1.33 (0.91-1.93)	2.05 (1.43-2.92)
	2	1.19 (0.82-1.73)	1.60 (1.10-2.34)
	3	1.14 (0.78-1.67)	1.50 (1.01-2.23)
**Total Cholesterol**	1	1.04 (0.73-1.48)	1.23 (0.87-1.73)
	2	1.20 (0.84-1.72)	1.55 (1.09-2.21)
	3	1.39 (0.96-2.02)	1.94 (1.30-2.88)
**Lp(a)**	1	1.06 (0.72-1.55)	1.41 (0.98-2.02)
	2	1.06 (0.72-1.57)	1.52 (1.02-2.26)
	3	1.09 (0.74-1.62)	1.44 (0.96-2.17)
**Triglycerides**	1	0.92 (0.64-1.31)	1.27 (0.91-1.77)
	2	0.99 (0.69-1.43)	1.49 (1.05-2.11)
	3	0.92 (0.64-1.34)	1.23 (0.83-1.81)
**LDL**	1	0.97 (0.68-1.38)	1.15 (0.82-1.62)
	2	1.08 (0.76-1.55)	1.41 (0.99-2.00)
	3	1.20 (0.83-1.74)	1.60 (1.09-2.35)
**GGT**	1	1.17 (0.81-1.69)	1.65 (1.16-2.34)
	2	1.16 (0.80-1.69)	1.77 (1.24-2.53)
	3	1.10 (0.76-1.60)	1.63 (1.14-2.35)
**Factor VIII**	1	1.00 (0.70-1.43)	1.43 (1.01-2.01)
	2	0.88 (0.62-1.27)	1.18 (0.83-1.67)
	3	0.88 (0.61-1.26)	1.11 (0.78-1.60)
**Galectin 3**	1	1.20 (0.83-1.73)	1.57 (1.09-2.25)
	2	1.11 (0.77-1.61)	1.37 (0.94-1.99)
	3	1.11(0.76-1.61)	1.23 (0.84-1.81)
**HDL**	1	1.03 (0.75-1.43)	0.81 (0.57-1.15)
	2	1.14 (0.82-1.59)	0.84 (0.58-1.21)

	3	1.15 (0.82-1.74)	0.84 (0.58-1.23)

* Model 1 was unadjusted.
Model 2 adjusted for age, sex, race, and region of residence.
Model 3 included tobacco use, total cholesterol, HDL, systolic blood pressure, hypertension treatment, diabetes, and use of statins, aspirin, and anticoagulation.
To analyze total cholesterol and HDL, each was eliminated from model 3 during its respective analysis.
When analyzing triglycerides and LDL, total cholesterol was excluded, as both lipoproteins contribute to total cholesterol levels.
Tertile cut points for each biomarker can be seen in supplementary table 1.

HR= hazard ratio. NTproBNP= N-terminal pro–B-type natriuretic peptide. GDF-15 = growth differentiation factor 15. GGT= Gamma-glutamyl transferase. LP(a)= lipoprotein (a). HLD= High-density lipoprotein, LDL= low density lipoprotein.

Table S5. Adjusted Hazard Ratio of Fatal and Non-fatal CHD per 1 SD In-Biomarker Increments in REGARDS Participants with Baseline Atrial Fibrillation *

Biomarker	Fatal CHD N=80	Non-fatal CHD N=121
**NTproBNP**	2.36 (1.80-3.09)	1.39 (1.11-1.75)
**GDF-15**	1.47 (1.09-1.99)	1.45 (1.15-1.82)
**C-reactive protein**	1.56 (1.22-2.00)	1.21 (0.99-1.49)
**Interleukin-6**	1.34 (1.12-1.60)	1.27 (1.07-1.49)
**D-dimer**	1.35 (1.08-1.70)	1.26 (1.03-1.54)
**Total Cholesterol**	1.20 (0.91-1.57)	1.34 (1.08-1.65)
**LP(a)**	1.30 (0.98-1.72)	1.27 (1.02-1.58)
**LDL**	1.18 (0.90-1.53)	1.28 (1.03-1.59)
**Triglycerides**	1.25 (0.96-1.62)	1.19 (0.97-1.46)
**GGT**	0.97 (0.74-1.25)	1.29 (1.07-1.55)
**Factor VIII**	1.03 (0.81-1.32)	1.14 (0.94-1.39)
**Galectin 3**	1.11 (0.85-1.44)	1.04 (0.85-1.28)
**HDL**	1.04 (0.82-1.31)	0.82 (0.67-1.00)

*Adjusted for age, sex, race, region of residence, included tobacco use, total cholesterol, HDL, systolic blood pressure, hypertension treatment, diabetes, and use of statins, aspirin, and anticoagulation. NTproBNP= N-terminal pro–B-type natriuretic peptide. GDF-15 = growth differentiation factor 15. GGT= Gamma-glutamyl transferase. LP(a)= lipoprotein (a). HDL= High-density lipoprotein, LDL= low density lipoprotein.

Table S6. Adjusted Hazard Ratio* of CHD per 1 SD In-Biomarker Increments in REGARDS Participants with Baseline Atrial Fibrillation by Anticoagulation Status.

	Irrespective of anticoagulation	Not on anticoagulation	Anticoagulated	P interaction
**NTproBNP**	1.69 (1.42-2.01)	1.67 (1.39- 2.00)	1.23 (0.87- 1.73)	0.05
**GDF-15**	1.45 (1.21-1.74)	1.46 (1.18- 1.80)	1.44 (1.01- 2.04)	0.22
**C-reactive protein**	1.33 (1.14-1.56)	1.26 (1.05- 1.51)	1.82 (1.32- 2.52)	0.05
**Interleukin-6**	1.29 (1.14-1.46)	1.28 (1.11- 1.47)	1.43 (1.09- 1.87)	0.56
**D-dimer**	1.29 (1.11-1.50)	1.35 (1.14- 1.60)	1.16 (0.85- 1.59)	0.08
**Cholesterol**	1.26 (1.07-1.49)	1.28 (1.07- 1.55)	1.22 (0.85- 1.75)	0.56
**Lp(a)**	1.26 (1.06-1.50)	1.27 (1.04- 1.55)	1.30 (0.92- 1.82)	0.93
**LDL**	1.21 (1.03-1.44)	1.24 (1.02- 1.50)	1.17 (0.83- 1.65)	0.85
**Triglycerides**	1.20 (1.02-1.41)	1.23 (1.03- 1.48)	1.10 (0.78- 1.57)	0.80
**GGT**	1.17 (1.01-1.37)	1.16 (0.98- 1.38)	1.21 (0.87- 1.68)	0.62
**Factor VIII**	1.10 (0.94-1.28)	1.08 (0.91- 1.28)	1.19 (0.87- 1.63)	0.77
**Galectin 3**	1.07 (0.91-1.26)	1.06 (0.88- 1.27)	1.21 (0.85- 1.70)	0.88
**HDL**	0.90 (0.78-1.05)	0.94 (0.80- 1.12)	0.78 (0.56- 1.09)	0.19

*Adjusted for age, sex, race, region of residence, included tobacco use, total cholesterol, HDL, systolic blood pressure, hypertension treatment, diabetes, and use of statins, aspirin, and anticoagulation. NTproBNP= N-terminal pro–B-type natriuretic peptide. GDF-15 = growth differentiation factor 15. GGT= Gamma-glutamyl transferase. Lp(a)= lipoprotein (a). HDL= High-density lipoprotein, LDL= low density lipoprotein.

Figure S1. Flow Diagram of the Cohort Selection Process.

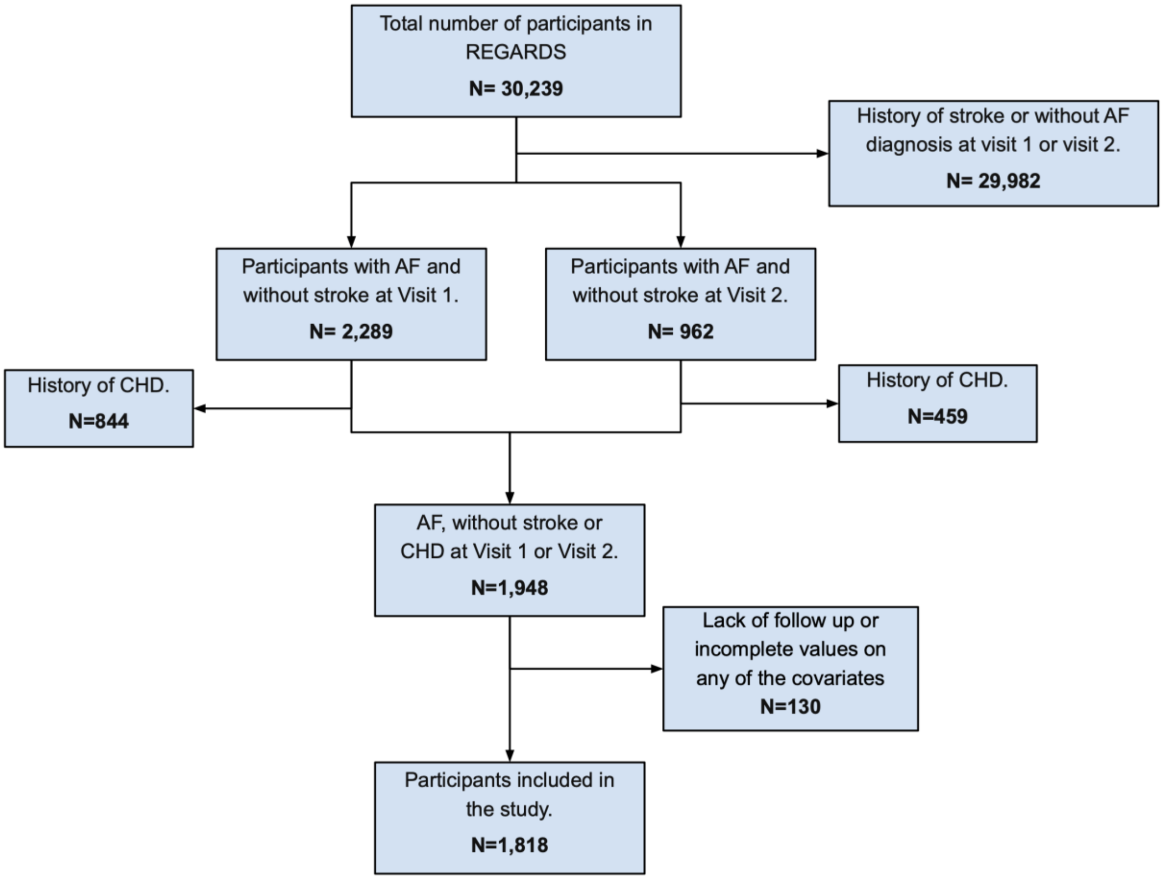

REGARDS= Reasons for Geographic and Racial Differences in Stroke. AF = atrial fibrillation. CHD= coronary heart disease.

Figure S2. Spline Plot Analysis of ln-transformed Biomarker Levels and CHD Risk. The HR (line) and 95% CI (blue shaded area) are shown in the top of each plot, with kernel density plots of biomarker distribution in the bottom of each plot.

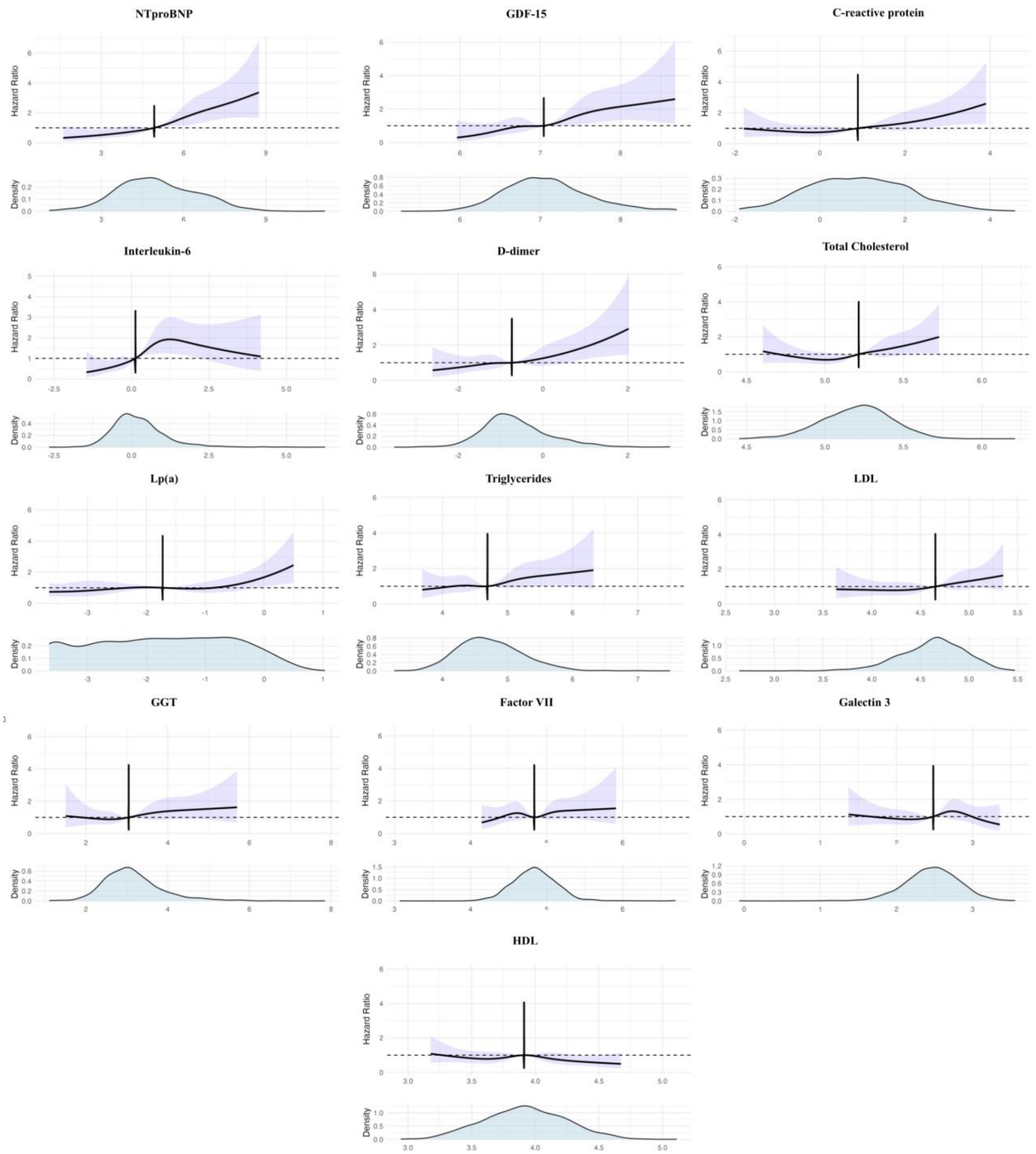

NTproBNP= N-terminal pro–B-type natriuretic peptide. GDF-15 = growth differentiation factor 15. GGT= Gamma-glutamyl transferase. LP(a)= lipoprotein (a). HLD= High-density lipoprotein, LDL= low density lipoprotein.
